# Precarious Work, Marital Quality, and Divorce: A Gendered Dyadic Analysis of Aging Couples

**DOI:** 10.1093/geroni/igaa057.2043

**Published:** 2020-12-16

**Authors:** Rachel Donnelly

**Affiliations:** Vanderbilt University, Nashville, Tennessee, United States

## Abstract

Precarious work – work that is unstable and insecure – is often stressful and may contribute to marital strain and dissolution among midlife adults. However, prior research has not considered how precarious work spills over to spouses. Using longitudinal dyadic data of midlife couples from the Health and Retirement Study, I examine whether having a spouse in precarious work is associated with marital strain and dissolution, with attention to differences by gender. I find that indicators of precarious work (job insecurity, schedule variability) are associated with a heightened risk of marital strain and divorce in midlife. These patterns depend on the gender of the spouse experiencing precarious work. Understanding the implications of precarious work for marriage is important because poor marital quality and divorce hasten health declines at older ages. Thus, this study suggests that precarious work may be a risk factor for divorce and poor health among aging adults.

